# Antibacterial activity of fresh pomegranate juice against clinical strains of *Staphylococcus epidermidis*

**DOI:** 10.3402/fnr.v59.27620

**Published:** 2015-05-20

**Authors:** Gabriel Betanzos-Cabrera, Perla Y. Montes-Rubio, Héctor E. Fabela-Illescas, Helen Belefant-Miller, Juan C. Cancino-Diaz

**Affiliations:** 1 Área Académica de Nutrición, Instituto de Ciencias de la Salud, Universidad Autónoma del Estado de Hidalgo, Hidalgo, Mexico; 2 Dale Bumpers National Rice Research Center, Stuttgart, AR, USA; 3 Departamento de Microbiología, Escuela Nacional de Ciencias Biológicas del Instituto Politécnico Nacional, Mexico City, Mexico

**Keywords:** pomegranate juice, Staphylococcus epidermidis, antimicrobial activity, antioxidant capacity, ocular infections

## Abstract

**Background:**

Polyphenols have received a great deal of attention due to their biological functions. Pomegranate (*Punica granatum* L.) is a polyphenol-rich fruit. In the past decade, studies testing the antimicrobial activity of pomegranates almost exclusively used solvent extracts instead of fresh pomegranate juice (FPJ). The use of FPJ instead of solvent extracts would reduce toxicity issues while increasing patient acceptance. We established a model to test FPJ as a natural antimicrobial agent.

**Objective:**

To evaluate the antimicrobial activity of FPJ on clinical isolates of multidrug-resistant *Staphylococcus epidermidis* strains.

**Design:**

Sixty strains of *S. epidermidis* isolated from ocular infections were grown in the presence of FPJ, and minimum inhibitory concentration (MIC) was determined by broth and agar dilution methods.

**Results:**

FPJ at 20% had a MIC equal to 100% (MIC_100%_) on all 60 strains tested. This inhibition of FPJ was confirmed by the growth kinetics of a multidrug-resistant strain exposed to different concentrations of FPJ. Additionally, the antimicrobial activity of FPJ was compared against commercial beverages containing pomegranate: Ocean Spray^®^ had a MIC_100%_ at 20%, followed by Del Valle^®^ with a MIC_15%_ at 20% concentration only. The beverages Jumex^®^ and Sonrisa^®^ did not have any antimicrobial activity. FPJ had the highest polyphenol content and antioxidant capacity.

**Conclusions:**

Overall, FPJ had antimicrobial activity, which might be attributed to its high polyphenol content and antioxidant capacity.

Pomegranate (*Punica granatum* L.) is the fruit of a tree belonging to the family Punicaceae. It is native from Iran to the Himalayas in northern India and has been cultivated and naturalized over the entire Mediterranean region since ancient times (1, 2). The ripe fruit contains many arils separated by a white, membranous pericarp (1). Studies show that pomegranate juice (PJ) has potent antioxidant activity (ability to scavenge free radicals), significantly higher than more commonly consumed fruit juices such as grape, cranberry, grapefruit, and orange (3–5). This activity has been attributed to antioxidant polyphenols, including ellagitannins (hydrolyzable tannins) and anthocyanins (condensed tannins) (6). Punicalagins are the major ellagitannins in the whole fruit and can be hydrolyzed to ellagic acid and other smaller polyphenols *in vivo* (1, 7).

Pomegranate has been used in traditional medicine for the treatment of dysentery, diarrhea, helminthiasis, and respiratory pathologies (8–11). Currently there is considerable interest toward evaluating plant sources for alternative treatments against multidrug-resistant pathogenic bacteria. The antimicrobial activity of pomegranate has been documented with different extracts (8, 12–15) and the potential therapeutic applications of the antimicrobial properties of pomegranate components have been investigated in human and murine models (1, 4, 16–21). However, FPJ antimicrobial activity has not been explored in spite of its high content of antioxidants.

There is a great interest in identifying antimicrobial compounds against *Staphylococcus epidermidis* because it, along with *S. aureus*, are public health problems due to their high incidence in hospitals. They also have a high capacity for resistance to antibiotics and other compounds, such as benzalkonium chloride and ethidium bromide, including inhibition of host immune responses (22). Their resistance to antibiotics is a result of their strong ability to produce biofilm. A biofilm is a multi-aggregate of microorganisms embedded in an exopolysaccharide with a high capacity for adherence to biotic and abiotic surfaces, protecting the bacteria from antibiotics and the immune system. The impact of infections by *S. epidermidis* has caused losses of $2 billion annually in the United States (23). Different commercial pomegranate-based beverages claim to have high antioxidant potency based only on their polyphenol content as determined by *in vitro* antioxidant assays (24). However, this does not necessarily indicate their biological activity and related health benefits. We compared FPJ activity with commercial pomegranate-based beverages for their ability to kill *S. epidermidis* and determined their total antioxidant activities. Antimicrobial activity has not been tested on pathogenic strains of *S. epidermidis*, so the aim of this work was to determine the antimicrobial activity of FPJ on clinical strains of multidrug-resistant *S. epidermidis*.

## Methods

### Fresh pomegranate juice

Pomegranates (*P. granatum* L.) were collected in Valle de Mezquital, Hidalgo, Mexico, in September 2012 and identified by Manuel González Ledesma, MSc, Universidad Autónoma de Hidalgo. A voucher specimen (U10) is deposited at the herbarium of the Department of Botany, Universidad Autónoma del Estado de Hidalgo, Mexico.

The fruit was handpicked, washed, and stored at 4°C. Fresh aril juice was used to ensure that the results would be valid for juice as consumed by humans. The fruit was peeled, and the arils were crushed and then squeezed with a household juicing appliance. The FPJ was sterilized by filtration with a 0.22 µm membrane (Millipore Corporation, Billerica, MA, USA) prior to use.

### Commercial pomegranate beverages

The four commercial beverages used for this study were those distributed nationally in Mexico and were purchased from a supermarket. All beverages were kept at storage conditions as specified on their labels prior to analysis. The beverages tested were 1) Del Valle^®^ ANTIOX (Batch 0471121631 13:42:57 CAD 16AGO11, Mexico D.F.); 2) Jumex^®^ SuperFrutas (Batch 2L B10J133 12:43 CAD 13OCT11, Mexico D.F); 3) Sonrisa^®^ Good 4 You (Batch 20510 CAD 24ENE12 Mexico D.F.); and 4) Ocean Spray^®^ Cranberry & Pomegranate (Batch CT8410209PP02 CAD 17MAY11 Lakeville-Middleboro, MA, USA).

### Bacterial strains, culture media, and growth conditions

Sixty strains of *S. epidermidis* isolated from human ocular infections (25, 26) were obtained from the Instituto de Oftalmología Conde de Valenciana, Mexico City. Their resistance to antibiotic and biocides are shown in Table 1. Half of the strains were resistant to multiple drugs and other compounds, such as ethidium bromide and benzalkonium chloride, and some of them were also biofilm producers. *S. epidermidis* strains were grown in Mueller Hinton broth (Becton Dickinson, Sparks, MD, USA) at 37°C for 24 h.

**Table 1:** *Staphylococcus epidermidis* strains with antibiotic and biocide resistance that were included in the study

Strains	Antibiotic resistance	Ocular infection	Biofilm production	MIC to EtBr (μg/mL)	MIC to BeCh (μg/mL)
51	Oxa, Tob, Cef, Cet, Pol, Sul, Amp	Conjunctivitis	Negative	ND	ND
60	Oxa, Ofl, Tob, Cef, Cet, Pol, Sul, Amp	Conjunctivitis	Positive	ND	ND
61	Oxa, Nor, Ofl, Tob, Cef, Pol, Sul, Amp	Conjunctivitis	Positive	ND	ND
63	Ofl, Tob, Nor, Gen, Cef, Cet, Tet, Pol, Sul	Endophthalmitis	Positive	50	8
71	Oxa, Tob, Cet, Tet, Pol, Amp	Conjunctivitis	Negative	100	4
93	Oxa, Cip, Ofl, Tob, Neo, Cef, Cet, Sul	Endophthalmitis	Positive	ND	ND
95	Oxa, Tob, Cef, Tet, Pol, Sul, Amp	Endophthalmitis	Positive	5	1
96	Tob, Cef, Pol, Sul, Amp	Endophthalmitis	Positive	>300	8
98	Oxa, Tob, Gen, Cef, Cet, Sul	Endophthalmitis	Negative	100	8
106	Oxa, Ofl, Tob, Gen, Cef, Cet, Sul	Conjunctivitis	Positive	ND	ND
144	Oxa, Ofl, Tob, Neo, Gen, Cef, Cet, Tet, Sul, Amp	Corneal ulcer	Positive	50	4
146	Oxa, Cip, Tob, Cef, Sul	Corneal ulcer	Positive	ND	ND
155	Pol, Sul	Conjunctivitis	Positive	5	1
199	Oxa, Nor, Ofl, Cef, Cet, Pol, Sul	Corneal ulcer	Negative	ND	ND
214	Oxa, Cip, Ofl, Tob, Neo, Gen, Nor, Cet, Sul	Endophthalmitis	Positive	100	4
215	Oxa, Tob, Neo, Gen, Pol, Sul, Amp	Conjunctivitis	Negative	100	8
1655	Cef, Cet, Pol	Conjunctivitis	Positive	10	2
1660	Tob, Cef, Cet, Pol, Amp	Conjunctivitis	Negative	10	2
1682	Oxa, Tob, Cep, Van, Sul	Conjunctivitis	Negative	100	8
1700	Gen, Tet	Conjunctivitis	Positive	10	2
1774	Oxa, Cef, Cet, Tet, Sul, Amp	Endophthalmitis	Negative	50	8
1784–2	Tob, Neo, Gen, Cet, Sul	Conjunctivitis	Positive	ND	ND
1819	Neo, Cef, Cet, Amp	Endophthalmitis	Negative	100	4
1843	Tob, Neo	Conjunctivitis	Positive	>300	8
1864	Cef, Amp	Corneal ulcer	Positive	ND	ND
1980	Tob, Tet, Sul, Amp	Endophthalmitis	Negative	ND	ND
2009	Cef, Amp	Endophthalmitis	Negative	100	8
2022	Oxa, Tob, Gen, Cef, Cet, Tet, Sul	Corneal ulcer	Negative	100	4
2038	Oxa, Ofl, Tob, Gen, Cef, Cet, Tet	Endophthalmitis	Positive	100	8
2050	Oxa, Cip, Ofl, Tob, Gen, Nor, Cef, Cet, Tet, Sul, Amp	Conjunctivitis	Negative	100	8

*Note*: The table only shows the 30 strains resistant to antibiotics of the 60 total studied.

Amp, ampicillin; Cef, ceftazidime; Cep, cephalothin; Cet, ceftriaxone; Cip, ciprofloxacin; EtBr, ethidium bromide; Gen, gentamicin; Neo, neomycin; Nor, norfloxacin; Ofl, ofloxacin; Oxa, oxacillin; Pol, polymyxin B; Sul, sulfisoxazole; Tet, tetracycline; Tob, tobramycin; Van, vancomycin; minimum inhibitory concentration (MIC)≥50 μg/mL is considered resistant. BeCh, benzalkonium chloride; MIC≥4 μg/mL is considered resistant. ND, not determined.

### Determination of MICs

The procedure to determine the MICs was performed according to the Clinical and Laboratory Standards Institute criteria (27). Briefly, for the broth dilution method, Mueller Hinton broth inoculated with ~10^4^ CFU ml^−1^ (adjusted to a turbidity of 0.5 McFarland) of each strain was supplemented aseptically with serial dilutions of FPJ or beverages to obtain concentrations from 0 to 20% (v/v), then the tubes were incubated at 37°C. MICs were determined visually at 24 and 48 h. For the agar dilution method, Petri dishes were prepared with Mueller Hinton agar plus the desired concentration of FPJ or commercial beverage. After hardening, the agar media were aseptically inoculated with 20 µL (~10^4^ CFU ml^−1^) of the tested bacterium. The lowest concentration of PJ or beverage capable of inhibiting visible growth after 48 h was recorded as the MIC. Each experiment was performed in triplicate.

### Growth curve of S. epidermidis

From an overnight culture, 20 µL of strain 144 (a bacterium selected for its biofilm production and multidrug resistance) were inoculated into Erlenmeyer flasks containing 50 ml of Mueller Hinton media supplemented with FPJ at the same concentrations used for the MIC. The cultures were incubated on a shaker at 37°C. The developing turbidity was measured using light absorption at 600 nm at time 0 and at 1 h intervals for 5 h. Uninoculated culture medium was used as blank.

### Total phenolic content

Total polyphenols were determined spectrophotometrically using the Folin–Ciocalteu method described by Singleton and Rossi (28). Total phenolics of FPJ and beverages were calculated and reported as gallic acid equivalents.

### Antioxidant assay – Trolox equivalent antioxidant capacity

Trolox equivalent antioxidant capacity (TEAC) was estimated as 2′,2′-azinobis(3-ethylbenzothiazline-6-sulfonic acid) diammonium salt (ABTS) radical scavenging activity according to the method of Re et al. (29). Briefly, ABTS (Sigma Aldrich, St. Louis, MO, USA) was dissolved in water to make a 7 mM concentration. ABTS radical cation (ABTS•^+^) was produced by reacting ABTS stock solution with 2.45 mM potassium persulfate (final concentration) and allowing the mixture to stand in the dark at room temperature for 12–16 h. The antioxidant standard was 6-hydroxy-2,5,7,8-tetramethylchroman-2-carboxylic acid (Trolox). A standard calibration curve was constructed for Trolox in ethanol (Sigma Aldrich) at 0, 50, 100, 150 and 200 mg/L. One hundred microliters of diluted samples were mixed with 900 µl of ABTS•^+^, and 200 µl of the mix were placed into 96-well plates, and absorbance at 734 nm was read after 5 min in a micro plate reader (Biotek^®^, Winooski, VT, USA). Samples were assayed in six replicates. TEAC values were calculated from the Trolox standard curve and expressed as Trolox equivalents (mg/L).

### Antioxidant assay free radical scavenging capacity

The free radical scavenging capacity was analyzed by 1,1-diphenyl-2-picrylhydrazyl (DPPH) assay according to the method of Morales and Jiménez-Pérez (30). Briefly, 100 µl of FPJ or beverages were mixed with 900 µl of a methanolic solution of 0.25 mM DPPH (Sigma Aldrich). The mixture was shaken and incubated at room temperature for 30 min, 200 µl were placed into 96-well plates, and the change in optical density at 520 nm was continuously monitored using a reader (Biotek^®^). Trolox was used as standard. Samples were assayed in six replicates. Percent inhibition was calculated from control using the following equation:

$$\text{Scavenging activity}(\%)=[(1-\text{Absorbanc}{\text{e}}_{\text{sample}}/\text{Absorbanc}{\text{e}}_{\text{control}}]\times \text{100}$$

### Statistical analysis

A one-way ANOVA with a Tukey test was used to analyze the results of bacterial growth kinetics, total phenolics, and antioxidant assays. The results were expressed as mean±SD. Differences were considered significant at *p*<0.05.

## Results

### Antimicrobial activity of FPJ in clinical strains of *S. epidermidis*

**Fig. 1:**
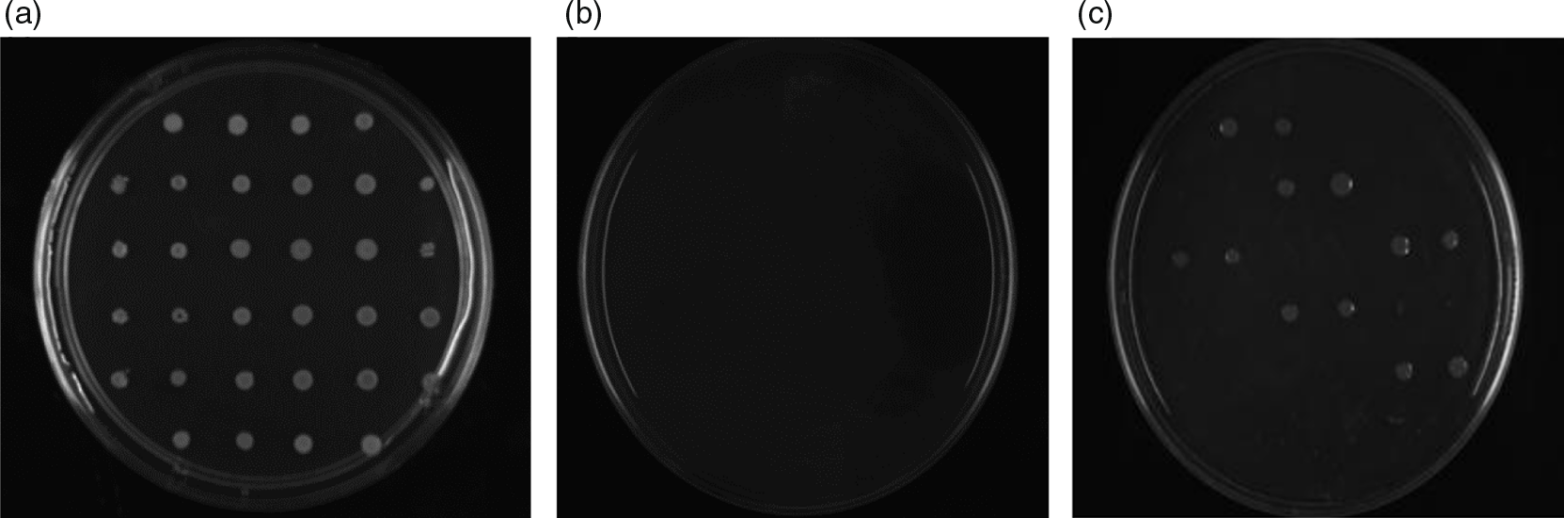
Antimicrobial activity of fresh pomegranate juice (FPJ). The following results are representative of all strains studied. Growth of 16 *S*. *epidermidis* strains (in duplicate), (a) unsupplemented, (b) supplemented with 20% FPJ, or (c) supplemented with 100 µg/mL of ampicillin. Note that six strains were resistant to this antibiotic.

Sixty clinical strains of *S. epidermidis* isolated from ocular infections were tested to observe the antimicrobial activity of FPJ. FPJ at 20% completely inhibited the growth of all 60 strains (Fig. 1a and b). Ampicillin was used as the control in measuring resistance to antimicrobial activity. Figure 1c shows various strains that were resistant to this ampicillin but were sensitive to FPJ at 20%.

Table 1 shows the phenotypes of 30 strains of *S. epidermidis* having resistance to multiple drugs, ethidium bromide, or benzalkonium chloride. In spite of their multiresistance, all these strains were sensitive to FPJ at 20%. The other 30 strains that were sensitive to antibiotics were also sensitive to FPJ and also were not biofilm producers (data not shown).

### Antimicrobial activity of commercial pomegranate-based beverages

**Fig. 2:**
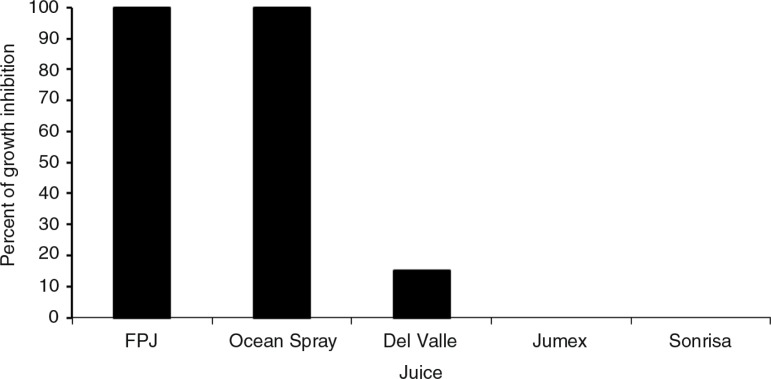
Percentage of growth inhibition of *S. epidermidis* by FPJ and beverages containing pomegranate. The results are for broth as well as agar. The concentration of each juice was 20%. Each assay was made in triplicate.

**Fig. 3:**
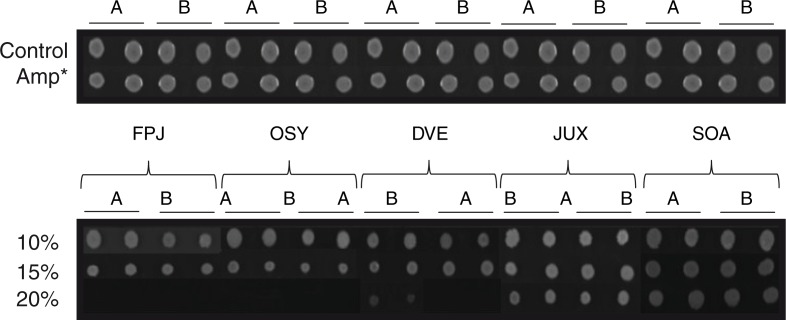
Comparison of antimicrobial activity between FPJ and commercial pomegranate-based beverages. An example of the effect of beverage concentration on strains 144 and 2050 (A and B, respectively) in duplicate. Control – Mueller Hinton media without FPJ; Amp – Mueller Hinton media containing 100 µg/mL of ampicillin; FPJ – fresh pomegranate juice; OSY – Ocean Spray^®^; DVE – Del Valle^®^; JUX – Jumex^®^; and SOA – Sonrisa^®^.

Figure 2 shows the antimicrobial activity of FPJ and various pomegranate beverages at 20% on *S. epidermidis* strains. FPJ and pomegranate beverages at less than 20% concentrations had no inhibitory effect. Both FPJ and Ocean Spray^®^ at 20% inhibited 100% of bacteria (MIC_100%_=20%), while Del Valle^®^ at 20% only inhibited 15% of the strains. Neither Jumex^®^ nor Sonrisa^®^ showed inhibitory effects at any concentration used (MIC_0%_=20%). The presence of a beverage, with the exception of Jumex^®^ and Sonrisa^®^, appeared to reduce the colony size (FPJ=Ocean Spray^®^>Del Valle^®^>Jumex^®^=Sonrisa^®^) (Fig. 3). It should be noted that adding FPJ or beverages to the culture medium did not alter the pH value (data not shown).

### Effect of FPJ on growth of *S. epidermidis* 144

**Fig. 4:**
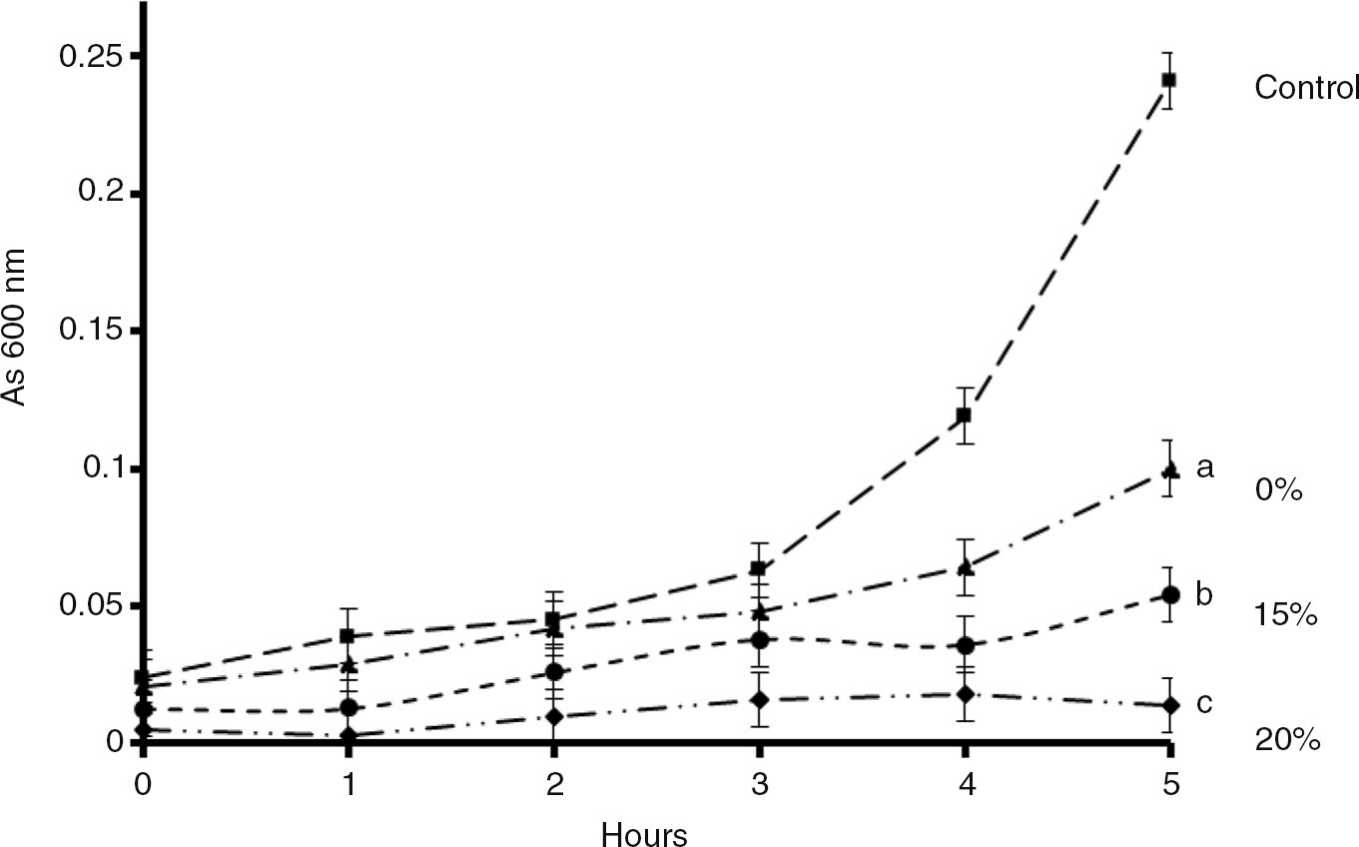
Effect of the concentration of FPJ on microbial growth of *S epidermidis* strain 144. Mueller Hinton broth was used for the growth curve. Control – growth without FPJ. Different letters represent significant differences (*p*<0.05) between the control and different concentrations (-▪- Control, -▴- 10%, -• -15%, -♦-20%) of FPJ according to a one-way ANOVA with a Tukey test.

In order to observe the effect of FPJ on the growth of the multiresistant and high biofilm producer strain 144, a bacterial growth curve was performed (Fig. 4). Even at low concentrations, the FPJ had an inhibitory effect on *S. epidermidis* growth while 20% FPJ completely inhibited growth for 5 h.

### Total polyphenol contents and antioxidant activity assays of FPJ and commercial pomegranate beverages

**Fig. 5:**
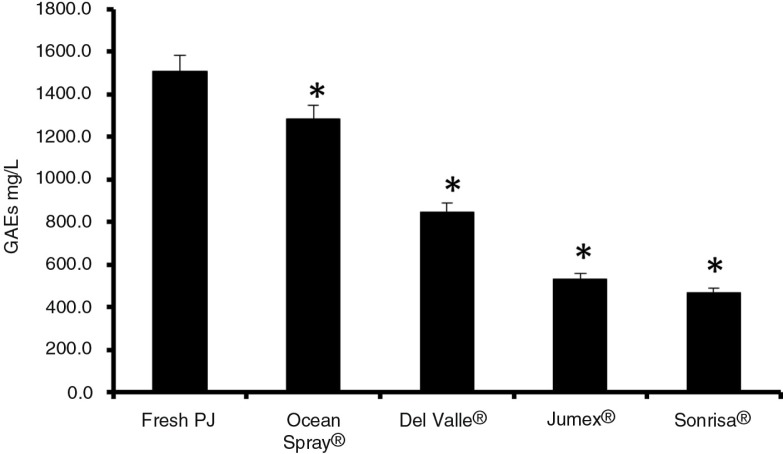
Total polyphenolics as gallic acid equivalents (GAE). Total polyphenol content was determined by the Folin-Ciocalteu method for all beverages. Fresh PJ – fresh pomegranate juice. An asterisk (*) indicates a significant difference (*p*<0.05) between FPJ and that beverage, according to a one-way ANOVA with a Tukey test.

**Fig. 6:**
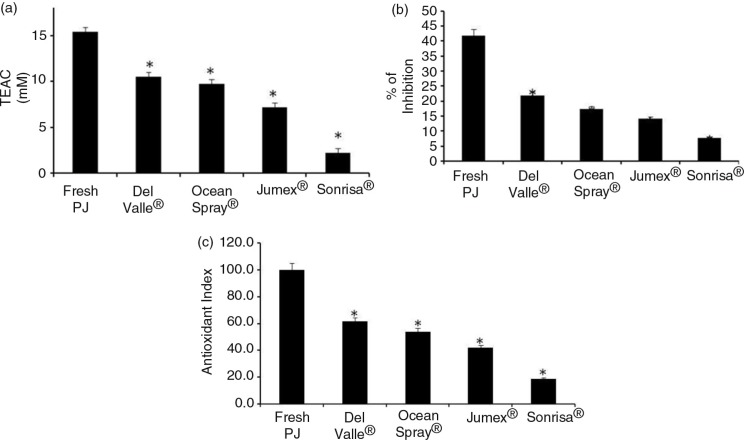
Antioxidant capacity of fresh pomegranate juice and pomegranate-based beverages. The antioxidant capacity was assayed by Trolox equivalent antioxidant capacity (TEAC) and 1-diphenyl-2-picrylhydrazyl (panels a and b, respectively). PJ – fresh pomegranate juice. Antioxidant index score=[(sample score/best score)×100] (panel c), average for all six tests for each beverage for the antioxidant potency composite index. An asterisk (*) indicates a significant difference (*p*<0.05) between FPJ and that beverage, according to a one-way ANOVA with a Tukey test.

The total polyphenol content of FPJ and pomegranate-based beverages occurred in the following order: FPJ>Ocean Spray^®^>Del Valle^®^>Jumex^®^=Sonrisa^®^ (Fig. 5). FPJ also had the highest antioxidant capacity in the two assays employed (Fig. 6a and b). When the measurements for polyphenols and antioxidants were combined into a single index of antioxidant activity (Fig. 6c), the rank order was the same as for the antimicrobial activity (Fig. 2).

## Discussion

*S. epidermidis* and *S. aureus* have acquired clinical importance because of their high prevalence in nosocomials and are found frequently in medical devices (31). Their infection capacity has been attributed to the production of biofilms and to their resistance to multiple antibiotics.

In this work, an MIC of 20% was observed for FPJ, which does not appear to indicate a strong antimicrobial effect. However, it should be taken into account that undiluted FPJ contains over 80% water. Therefore, a higher volume is necessary in order to produce a strong antimicrobial effect, unless concentrate or extracts are obtained. This work cannot be directly compared to others that used organic solvent extracts, because FPJ is only derived from the arils, whereas many organic extracts use other parts of the fruit, that is, the peel, whole fruit, or rind. Concentration and volume differences would also make comparisons difficult. Several extracts and concentrates have been demonstrated to have antimicrobial activity against a variety of bacteria, for example: methanolic extracts of the fruit rind (32); chloroform, ethanol, and water extracts of the pomegranate plant (33); pomegranate fruit liquid extracts (ethanolic and butanolic) (10); methanolic extract of pomegranate peels (12); acetone, methanol, ethanol, and water extracts from pomegranate (34); and ethanolic extract of pomegranate peel (9). However, we suggest that FPJ would be a historically safe, convenient, and highly palatable method with similar results to those of extracts.

Several compounds responsible for the antimicrobial action of each pomegranate beverage, depending on their abundance. One example is tannins (abundant in pomegranate), which are considered to be toxic to microorganisms (35). Their hydrophilic part interacts with the polar region of the membrane, whereas the hydrophobic part is immersed in the non-polar inner region of the bacterial membrane, which can cause instability of the membrane, thus affecting the transport of substrates into the cell (36). Likewise, Naz et al. (37) suggest a phenolic toxicity through reactions with sulfhydryl groups or through more non-specific interactions with proteins leading to loss of function.

On the other hand, phenols may also render substrates unavailable to microorganisms or interfere with bacterial protein secretions (24). Additionally, high-performance liquid chromatography analyses have shown that the major phenolic compounds in pomegranate are gallic acid and ellagic acid, in addition to punicalagin as a major ellagitannin. Thus, it is reasonable to consider that these compounds may be responsible for the antimicrobial activity, as suggested by Ahn et al. (38). However, it might be possible that other unidentified compounds contribute to this activity.

It is well known that industrialized food loses some of its properties compared to unprocessed food. Pomegranate-based industrialized beverages were tested to find if they keep or lose antibacterial activity relative to fresh juice. Although Ocean Spray^®^ beverage had the same MIC as FPJ, Del Valle^®^ had only 15% antibacterial activity, whereas Jumex^®^ and Sonrisa^®^ beverages did not have any (Fig. 2). Ocean Spray had the same antibacterial activity compared to FPJ. However, it should be noted that this beverage's label states that it contains not only pomegranate concentrate, but also a variety of other fruit concentrates, including grape, apple, plum, and cranberry, which may have contributed to the antibacterial activity. Commercial products commonly claim to have antioxidant potency from a high content of polyphenols; however, this claim is usually based only on a limited number of antioxidant tests. When we applied two tests of antioxidant potency, TEAC and DPPH, and calculated an overall antioxidant potency composite index, we saw slight differences between individual polyphenol measurements; however, when combined into an antioxidant index, the rankings matched the percent inhibition. The results of antioxidant capacity were consistent with Seeram et al. (24), who used four methods, including the two tested in this work, and demonstrated that the compounds have different abilities as antioxidants and therefore calculating an antioxidant potency composite index is recommended in order to get a reliable measurement.

Clinical *S. epidermidis* strains are a good test organism for evaluating antibacterial activity because of the high genomic variation in this microorganism, even to being considered a microorganism with an open pangenome (23). In this work, FPJ exhibited antibacterial activity against 60 strains of *S. epidermidis*. A future challenge will be to test the antimicrobial activity of FPJ against other microorganisms of medical importance including fungi, viruses, and bacteria isolated from foods; we expect to find the same effect with FPJ because previous reports have demonstrated such activity using extracts (10, 12, 32–34).

In human and murine models, PJ has been shown to exert several potential health benefits, including antihypertensive (16–18, 39, 40), antiatherosclerotic (16, 20), and anti-inflammatory effects (21, 41, 42). Extracts or commercial beverages may contain a wide range of synthetic antimicrobial compounds, such as sodium benzoate, sorbate, and synthetic antioxidants, that are used as food preservatives but can be harmful (43), so FPJ may be more beneficial.

## Conclusions

The antimicrobial activity of FPJ on pathogenic bacteria described here could provide an alternative natural antibacterial treatment, although further studies should be conducted on a wide variety of bacteria and direct relationships established between antimicrobial activity and polyphenols. Identification of the antimicrobial agents in PJ could also yield valuable information.
